# Soil microbial diversity and functional capacity associated with the production of edible mushroom *Stropharia rugosoannulata* in croplands

**DOI:** 10.7717/peerj.14130

**Published:** 2022-10-03

**Authors:** Shaojun Tang, Tingting Fan, Lei Jin, Pin Lei, Chenxia Shao, Shenlian Wu, Yi Yang, Yuelin He, Rui Ren, Jun Xu

**Affiliations:** 1 Hunan Institute of Microbiology, Changsha, china; 2 College of Forestry, Central South University of Forestry & Technology, Changsha, China

**Keywords:** Soil physicochemical properties, *Stropharia rugosoannulata*, Soil bacterial communities, Soil fungal communities, Functional analysis

## Abstract

In recent years, a rare edible mushroom *Stropharia rugosoannulata* has become popular. *S. rugosoannulata* has the characteristics of easy cultivation, low cost, high output value, and low labor requirement, making its economic benefits significantly superior to those of other planting industries. Accumulating research demonstrates that cultivating edible fungus is advantageous for farming soil. The present experiment used idle croplands in winter for *S. rugosoannulata* cultivation. We explored the effects of *S. rugosoannulata* cultivation on soil properties and soil microbial community structure in paddy and dry fields, respectively. We cultivated *S. rugosoannulata* in the fields after planting chili and rice, respectively. The results showed that Chili-*S. rugosoannulata* and Rice-*S. rugosoannulata* planting patterns increased the yield, quality and amino acid content of *S. rugosoannulata*. By analyzing the soil properties, we found that the Chili-*S. rugosoannulata* and Rice-*S. rugosoannulata* cropping patterns increased the total nitrogen, available phosphorus, soil organic carbon, and available potassium content of the soil. We used 16s amplicons for bacteria and internal transcribed spacer (ITS) region for fungi to analyze the microbial communities in rhizosphere soils. Notably, *S. rugosoannulata* cultivation significantly increased the abundance of beneficial microorganisms such as *Chloroflexi*, *Cladosporium* and *Mortierella* and reduce the abundance of *Botryotrichumin* and *Archaeorhizomyces*. We consider *S. rugosoannulata* cultivation in cropland can improve soil properties, regulate the community structure of soil microorganisms, increase the expression abundance of beneficial organisms and ultimately improve the *S. rugosoannulata* yield and lay a good foundation for a new round of crops after this edible mushroom cultivation.

## Introduction

The edible fungi industry is an important industry in the global planting industry, and most edible fungi extracts have biological activities such as antioxidants (Pejin et al., 2019; Tesanovic et al., 2017). *S. rugosoannulata* is a rare edible mushroom originally discovered by Professor Murrill in the United States (Yan et al., 2020). Because of its high economic and ecological value, *S. rugosoannulata* was introduced to China as early as the 1980s and has been widely planted (Yan et al., 2020). Experimental pharmacological studies have shown that *S. rugosoannulata* has various biological activities such as antitumor, antibacterial, antioxidant, and immunomodulatory (Wu et al., 2011; Wu et al., 2012). In recent years, the demand for *S. rugosoannulata* has been increasing due to its delicious and pharmacological properties, so it is urgent to increase its production  (Liu et al., 2020).

Crop rotation appears to be an effective method for boosting *S. rugosoannulata* output (Peltonen-Sainio, Jauhiainen & Lehtonen, 2016). This is due to long-term inappropriate use of the land, such as diffuse irrigation, excessive use of fertilizers, pesticides, etc., can lead to damage to the soil structure, imbalance of nutrient ratios, degradation, and acidification  (Gong et al., 2018). Rotation can increase the soil organic carbon and total nitrogen content, improve soil fertility, and boost crop output (Kuht et al., 2019; Eremeev et al., 2020). The mycelium produced by growing edible mushrooms contains various trace elements (Koutrotsios et al., 2020). The residual mycelium is rich in crude protein, crude fat, calcium, phosphorus, and other organic substances (Yun et al., 2021). Some bioactive substances secreted by the mycelium in the production process can decompose complex organic matter, inhibit some soil-borne diseases and promote plant growth (Grimm & Wösten, 2018). The texture of the mushroom residue is loose and has a solid ability to absorb water, which can be further decomposed into humus with good air and water storage capacity and can effectively improve the soil (Lipiec et al., 2021). This indicates that mushroom residue can well regulate the soil properties of agricultural land and enhance soil fertility. In addition, crop cultivation in cropland also contributes to mushroom culture (De la Fuente, Beyer & Rinker, 1998). That is because the rotation of different crops on the same soil can significantly improve the physical and chemical properties of the soil, regulate soil fertility, and ultimately achieve increased production and income (Kumar et al., 2020).

Chili and rice are two important economic crops in China’s Hunan Province, and both have a positive impact on soil quality. Therefore, it seems to be a good choice to cultivate *S. rugosoannulata* in winter on unused acreage. Studies have indicated that using chili as a crop rotation can greatly enhance soil nitrogen content and minerals after planting, as well as increase carbon fixation in soil (Hao et al., 2011). Hahm et al. (2017) discovered that using chili as a crop rotation boosted the expression abundance of *Chloroflexi* in soil while decreasing salt accumulation. In addition to regulating soil microorganisms, chili inhibits the development and proliferation of *Meloidogyne* spp., according to research conducted by Bafokuzara (1983). Some experiments have found that the implementation of paddy upland rotations increases the stability of soil physical properties, improves the bulk density of soil, reduces damage to soil structure, and increases soil water stable aggregate (Hou et al., 2018). On the other hand, unlike dryland soils alone, soils in paddy upland rotations change the chemical form of the nutrients in them due to alternating wet and dry, anaerobic and aerobic conditions (Zhou et al., 2014). Crop rotation can effectively improve soil utilization, improve soil structure through the roots of different crops, reduce soil consolidation and degradation, and increase soil nutrient content (Hamza & Anderson, 2005).

Currently, the majority of research use horticultural crops in rotation with chili or rice. Few studies have investigated the influence of edible fungus as rotation crops on soil characteristics. In this experiment, *S. rugosoannulata* was used for the first time to rotate with chili and rice. Additionally, *S. rugosoannulata* was cultivated by maximizing the use of idle farmland during the winter, and it was investigated whether the cultivation process as a whole could regulate soil properties and soil microorganisms. This study will contribute to the commercial production of *S. rugosoannulata* and enhance the utilization of vacant agricultural land in winter.

## Materials & Methods

### Experimental design

Three experimental plots of the same size (two hectare) in the experimental field of the Hunan Institute of Microbiology (longitude: 112.98, Latitude: 28.12) were selected. These three plots have the same size and adjacent positions. The experiment began in April 2020. The chili variety used in the experiment was (*Capsicum annuum L.*), ‘SJ11-3’. The rice variety used in the experiment was “Zhongjiazao 17”. Cultivation of *S. rugosoannulata* in vacant fields (NC), cultivated *S. rugosoannulata* after harvested chili (Chili-*S. rugosoannulata*) and rice (Rice-*S. rugosoannulata*) crops. In April 2020, chili was planted in the experimental field of the Chili-*S. rugosoannulata* group. Chili was harvested in October 2020. In June, rice was planted in the experimental field of the Rice-*S. rugosoannulata* group. Rice was harvested in September 2020. In mid-November 2020, the ground of all test fields was leveled and all crop residues were removed and not plowed into the soil. The quicklime was used to disinfect and kill insects on the fields. Straw, corncob, and chaff was soaked in 3% lime water for two days, then mixed in an 8:1:1 ratio and distributed equally with a thickness of 30 cm on all experimental fields. It was then paved with the *S. rugosoannulata* spawn (300 kg/mu) and covered with approximately 2–3 cm of soil. After covering the soil, it was covered with a layer of rice straw (the covering layer is not visible, the thickness is 3–4 cm). Three to four months were required for the development of the fungi to fruiting bodies. The root soil from the three experimental fields was sampled the same year in mid–November, next year in January and March to see the effect of fungal growth on the soil quality. We used the five-point sampling method to produce a composite sample by combining five independent soil cores from around and within the croplands (Liu et al., 2021a). Six composite samples were taken from each experimental field. Fresh samples were sealed in plastic bags and stored in a −80° freezer.

### Determination of amino acid content in *S. rugosoannulata*

After crushing *S. rugosoannulata* through a 50-mesh sieve (the aperture is 0.3 mm), add 2 ml of concentrated hydrochloric acid, mix 2 to 3 drops of phenol and evacuate heat melt the glass hydrolysis tube while keeping the hydrolysis tube in a high vacuum state, and complete the sealing. Place the hydrolysis tube in an oven at 110 °C. After hydrolyzing for 24 h, take it out and cool it to room temperature. Transfer the hydrolyzed sample to a 25ml volumetric flask. Wash the hydrolysis tube repeatedly with pure water and fill the volume up to the mark. After the constant volume of the sample is passed through a 0.22 µm microporous membrane, put it into the sample vial to be tested. Analysis and identification of amino acids in biological tissues utilizing a high-performance liquid chromatograph (Model 1100; Agilent, USA). The chromatographic conditions are: chromatographic column: Zorbax Eclipse AAA C18 column (75 mm × 4.6 mm, 3.5 µm, Agilent, USA); mobile phase: A is 40 mmol/L NaH_2_PO_4_ solution, pH 7.8; B is acetonitrile-methanol-water (volume ratio of 4.5:4.5:1). Mobile phase gradient elution program: 0.0–1.0 min (B: 0%), 1.0–9.8 min (B: 0%–57%), 9.8–10.0 min (B: 57% ∼100%), 10.0–12.0 min (B: 100%), 12.0–12.5 min (B: 100% ∼0%), 12.5–14.0 min (B: 0%); flow rate: two mL/min; column temperature: 40 °C; injection volume: 18 µl.

### Determination of soil total nitrogen

A total of 1.00 g air-dried soil sample with 100-mesh sieve (the aperture is 0.15 mm) was weighed and carefully placed in the Kjeldahl flask. A small amount of distilled water was added for wetting and washing. Then, 2 g of the prepared mixed catalyst was added, and 5 ml of concentrated sulfuric acid was added to cover the bottle cap with a small funnel. Place the prepared bottle on the digester at 375 °C for 1 h. The end of cooking is a clear light blue mixture of the cooking furnace. After digestion, the Kai-type bottle was removed, standing for cooling, shaking in the middle for several times to prevent precipitation, and the small funnel was taken down. The small funnel was washed with distilled water to make all the solution in the small funnel enter the Kai-type bottle. The supernatant was taken and diluted to adjust the pH value. The total nitrogen content in the solution was measured by a flow analyzer (AA3Auto Analyzer 3 Continuous-Flow Analysis-3; SEAL, Germany), and then converted into soil total nitrogen content (Dane, Topp & Campbell, 2002).

### Determination of soil available phosphorus

Weigh 2.5 g air-dried soil sample which has passed through a 20-mesh sieve (the aperture is 0.85 mm), add 50 ml NaHCO_3_ (0.5 mol L^−1^) solution and phosphorus-free activated carbon, shake for 30 min, and filter. Remove the filtrate and add 2-6 dinitrophenol color reagent, adjust the solution to slightly yellow with diluted acid, add Mo-Sb antimony reagent and read the absorbance value at 720 nm wavelength (Bray & Kurtz, 1945).

### Determination of soil organic carbon

Weigh a soil sample that has been quantitatively passed through an 80-mesh sieve (the aperture is 0.18 mm) placed in a dry hard test tube, with the transfer tube to accurately add 0.8000 mol/L (1/6K_2_CrO_7_) standard solution 5 mL, accurately add concentrated sulfuric acid 5 ml fully shake. The test tube was placed in a wire cage, and then the wire cage was placed in an oil bath to heat. After being placed, the temperature should be controlled at 170–180 °C. When the liquid in the test tube boils and bubbles occur, it starts to count, boils for 5 min, removes the test tube, slightly cools, and wipes out the external oil of the test tube. Take out the cooling. After cooling, all the contents in the test tube were carefully transferred into 250 ml triangle bottle, so that the volume of the contents in the triangle bottle was 60–70 ml. Keep sulfuric acid concentration of 1∼1.5 mol/l, the solution color should be orange or light yellow. Then add o-norphine indicator 3∼4 drops, titrate with 0.2 mol/l standard ferrous sulfate (FeSO_4_) solution, the solution from yellow through green, light green to brown is the end point (Ciavatta et al., 1991).

### Determination of soil moisture

Weigh a 5 g soil sample, put it in an oven at 105 °C, and bake it until the weighing remains constant before and after twice. Loss of moisture after drying is the soil moisture content.

### Determination of soil available potassium

Weigh a 5 g air-dried soil sample which has passed through a 20-mesh sieve (the aperture is 0.85 mm), add 50 ml NH_4_OAc (1 mol L^−1^) solution, shake for 30 min, filter, and take the filtrate for measurement on a flame photometer (Jackson, 2005).

### Soil DNA extraction and library preparation

Use EZNA® soil DNA kit (Omega Bio-tek, Norcross, GA, USA) to extract DNA from soil samples. The primer pair 338F (5′-ACTCCTACGGGAGGCAGCA-3′) and 806R (5′-GGACTACHVGGGTWTCTAAT-3′) was used to amplify the bacterial 16S V3+V4 region of extract DNA. The fungal ITS2 region of extract DNA was amplified using primer pair ITS2F (5′-GCATCGATGAAGAACGCAGC-3′) and ITS2R (5′-TCCTCCGCTTATTGATATGC-3′). PCR reaction conditions were 25 cycles of 95 °C 5min, 95 °C 30 s, 50 °C 30 s, 72 °C 40 s, and finally 72 °C for 7 min, and stored at 4 °C. The PCR products obtained were quantified by electrophoresis (ImageJ software) and mixed in a mass ratio of 1:1. Column purification was performed using OMEGA DNA purification columns (bio-tek, Doraville, GA, USA). Finally, use 1.8% agarose gel, electrophoresis at 120 V for 40min, and cut the gel to recover the target fragment.

### Illumina sequencing and microbial diversity analysis

The samples were sequenced on the Illumina MiSeq platform, the original data were spliced (FLASH, version 1.2.11) (Magoč & Salzberg, 2011), using Trimmomatic (version 0.33) to filter the quality of the raw data (Bolger, Lohse & Usadel, 2014), then use Cutadapt (version 1.9.1) to identify and remove primer sequences, and then use USEARCH (version 10) to splice paired-end reads and remove chimeras (UCHIME, version 8.1) finally obtained high-quality sequences for subsequent analysis (Edgar et al., 2011). Sequences with a similarity of more than 97% were clustered as an OTU (USEARCH, version 10.0), and 0.005% of all sequences sequenced were used as a threshold to filter OTUs (Edgar, 2013). Bacteria were annotated using the Silva database (Release128, http://www.arb-silva.de), and fungi were annotated using Unite (Release 7.2, http://unite.ut.ee/index.php). Species annotation was performed using RDP Classifier with a confidence threshold of 0.8 (version 2.2, https://sourceforge.net/projects/rdp-classifier/). Mothur version 1.30 (http://www.mothur.org/) for Alpha diversity index analysis. All sample sequences were uploaded to the NCBI database, with sample numbers ranging from SRX13373992 to SRX13374011.

### Correlation and predictive analysis of microbial function

Correlation analysis and mapping of soil quality and soil microorganisms using the R language vegan (v2.3) package. RDA (Redundancy analysis) is a sorting method developed based on correspondence analysis; RDA analysis is based on a linear model, mainly used to reflect the relationship between flora or samples and environmental factors. The size of the first axis of Lengths of a gradient in the analysis result should be less than 4.0. PICRUSt2 (Phylogenetic Investigation of Communities by Reconstruction of Unobserved States 2) software was used to predict the abundance of marker functional gene sequences in samples. PICRUSt2 is a computational method that utilizes marker gene data and a reference genome database to predict the functional composition of environmental microbes, based on IMG microbial genome data to predict microbial communities’ functional potential during phylogeny through phylogeny phylogenetic-functional correlations. Based on the software’s reference genome data, both 16S rRNA sequences and ITS sequences can be used for functional prediction (Langille et al., 2013).

### Determination of *S. rugosoannulata* quality

Take the fruiting body of *S. rugosoannulata*, measure the diameter and length of its stipe, and determine whether it is hollow. 5 kg of fruiting bodies were randomly selected in each experimental field to evaluate the quality of *S. rugosoannulata*.

### Data analysis

All data in this study were analyzed using SPSS 22.0, expressed as mean  ± standard deviation of the mean (SD). The difference between the means of the groups was analyzed using a one-way ANOVA pair and evaluated using Tukey multiple comparisons. *p* < 0.05 was regarded as a significant difference.

## Results

### Quality and yield of *S. rugosoannulata* in different croplands

**Figure 1:**
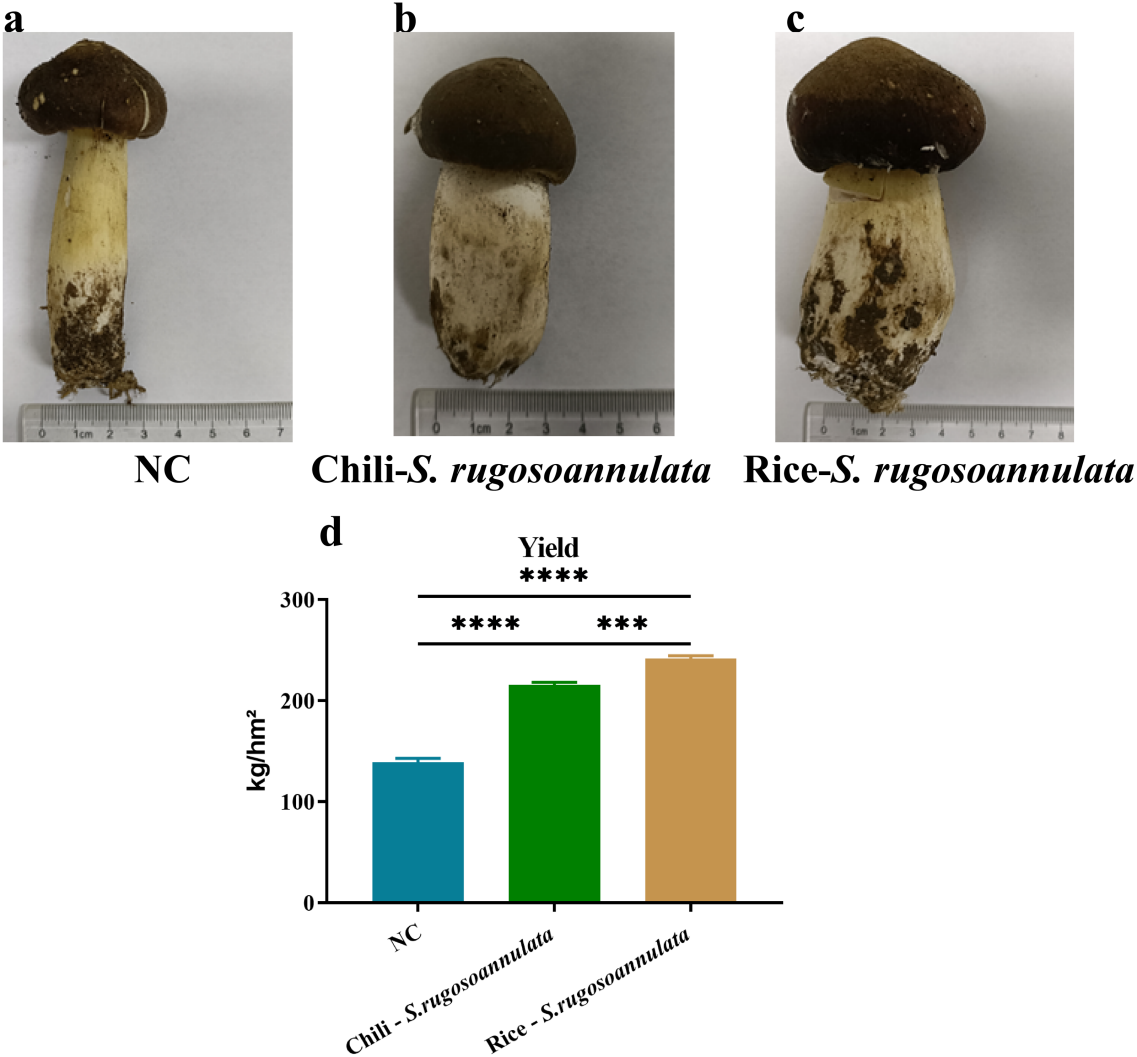
The quality and yield of *S. rugosoannulata* in different croplands. (A) *S. rugosoannulata* produced in the NC group; (B) *S. rugosoannulata* produced in the Chili-*S. rugosoannulata* group; (C) *S. rugosoannulata* produced in the Rice-*S. rugosoannulata* group; (D) Yield per mu of *S. rugosoannulata* in different croplands. Data are mean ± SD (*n* = 3) and analyzed by one-way ANOVA. *p < 0.05.

First, we determined the quality of *S. rugosoannulata*. As shown in Fig. 1, the mushroom stipe of *S. rugosoannulata* grown in the NC group was elongated and hollow (Fig. 1A). However, compared with the NC group, the stipe of *S. rugosoannulata* grown in rotation with chili and rice was significantly thicker (Figs. 1B and 1C). Among them, *S. rugosoannulata* which was rotated with rice, has the thickest mushroom stipe, the largest volume, and the best quality. The yield of *S. rugosoannulata* in different croplands was shown in Fig. 1D. Compared with NC, the yield of Chili-*S. rugosoannulata* and Rice-*S. rugosoannulata* was significantly higher (*p* < 0.05). Among them, *S. rugosoannulata*, which was rotated with rice, has the highest yield. It reached about 240 kg/hm^2^.

### Amino acid content of *S. rugosoannulata* in different croplands

Table 1 shows the analysis of amino acid content in *S. rugosoannulata*. For the *S. rugosoannulata* in Chili-*S. rugosoannulata*, there was a substantial increase in the level of Thr, Ser, Glu, Gly, Ala, Val, Ile, Leu, Tyr, His, Lys, and Pro (*p* < 0.05) relative to the control *S. rugosoannulata.* Meanwhile, for the *S. rugosoannulata* in Rice-*S. rugosoannulata*, the levels of 16 types of amino acids increased substantially (*p* < 0.05) in comparison to the control *S. rugosoannulata*.

**Table 1:** The amino acid content of *S. rugosoannulata* in different croplands.

Amino acid (µg/mg)	NC	Chili-*S. rugosoannulata*	Rice-*S. rugosoannulata*
Asp	1.670 ± 0.039^b^	1.589 ± 0.053^b^	2.060 ± 0.041^a^
Thr	0.901 ± 0.023^c^	0.968 ± 0.017^b^	1.128 ± 0.050^a^
Ser	0.892 ± 0.025^c^	0.984 ± 0.024^b^	1.091 ± 0.038^a^
Glu	2.362 ± 0.222^b^	3.231 ± 0.036^a^	3.067 ± 0.085^a^
Gly	0.715 ± 0.018^c^	0.813 ± 0.029^b^	1.071 ± 0.021^a^
Ala	0.975 ± 0.008^c^	1.367 ± 0.102^b^	1.502 ± 0.030^a^
Val	0.972 ± 0.021^c^	1.104 ± 0.034^b^	1.252 ± 0.022^a^
Met	0.313 ± 0.019^b^	0.308 ± 0.020^b^	0.480 ± 0.042^a^
Ile	0.667 ± 0.021^c^	0.801 ± 0.015^b^	0.905 ± 0.022^a^
Leu	1.225 ± 0.038^b^	1.487 ± 0.196^a^	1.557 ± 0.053^a^
Tyr	0.538 ± 0.022^b^	0.620 ± 0.020^a^	0.594 ± 0.015^a^
Phe	0.855 ± 0.009^b^	0.940 ± 0.023^b^	1.043 ± 0.082^a^
His	2.967 ± 0.085^c^	2.235 ± 0.035^b^	5.881 ± 0.159^a^
Lys	0.960 ± 0.025^c^	1.078 ± 0.031^b^	1.388 ± 0.026^a^
Arg	0.746 ± 0.015^b^	0.783 ± 0.016^b^	0.921 ± 0.036^a^
Pro	0.486 ± 0.015^b^	0.558 ± 0.037^a^	0.599 ± 0.015^a^

**Notes.**

a,b,c Mean values with different letters are significantly different from each other (*p* < 0.05).

### Soil properties of different croplands

**Figure 2:**
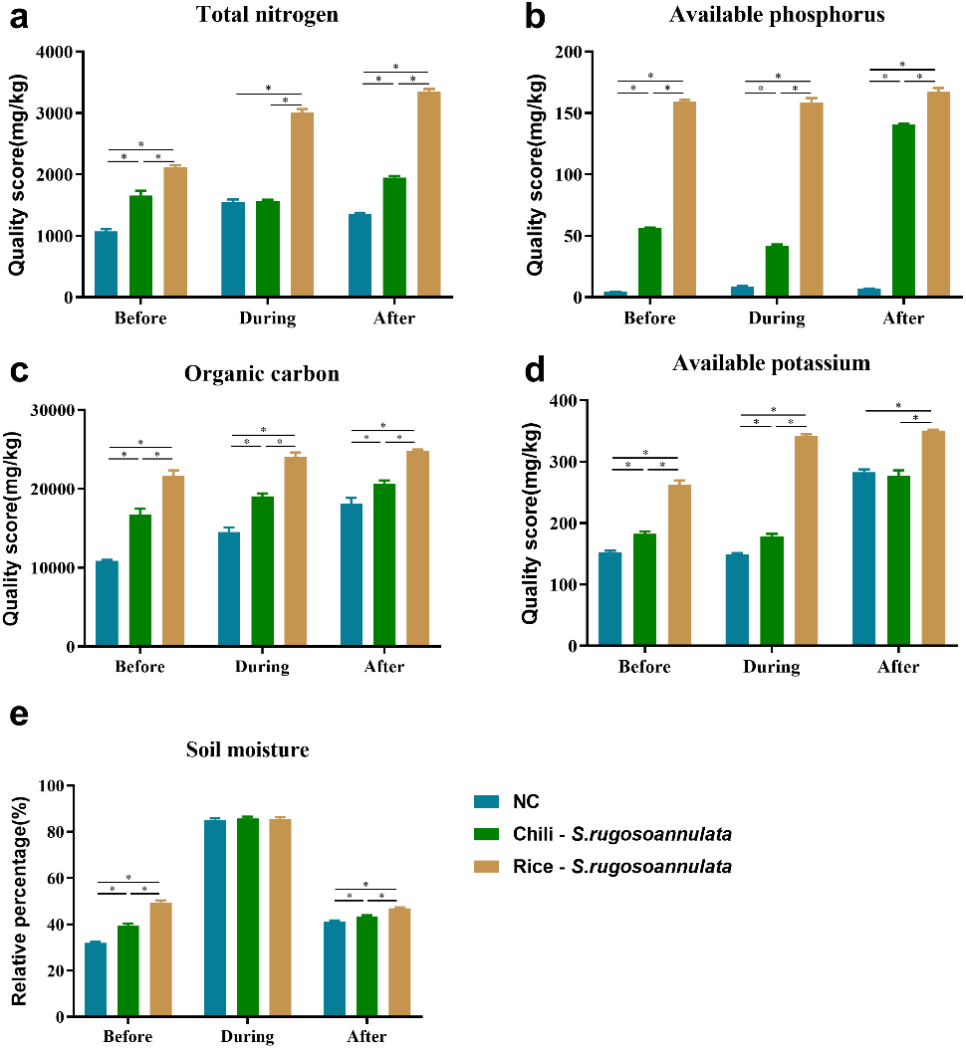
Soil properties in different croplands. (A) The total nitrogen content of Soil before, during, and after cultivating *S. rugosoannulata* under different rotation patterns; (B) The available phosphorus content of Soil before, during, and after cultivating *S. rugosoannulata* under different rotation patterns; (C) The organic carbon content of Soil before, during, and after cultivating *S. rugosoannulata* under different rotation patterns; (D) The available potassium content of Soil before, during, and after cultivating *S. rugosoannulata* under different rotation patterns; (E) The soil moisture before, during, and after cultivating *S. rugosoannulata* under different rotation patterns. Data are mean ± SD (*n* = 3) and analyzed by one-way ANOVA. *p < 0.05.

We found that compared with the NC control group, the Chili-*S. rugosoannulata* and Rice-*S. rugosoannulata* rotations significantly improved the total nitrogen (Fig. 2A), available phosphorus (Fig. 2B), soil organic carbon (Fig. 2C) and available potassium (Fig. 2D) content in the soil before, during and after cultivating *S. rugosoannulata* (*p* < 0.05). Among them, the soil of *S. rugosoannulata* and rice rotation has the highest content of total nitrogen, available phosphorus, soil organic carbon, and available potassium. Compared with the NC control group, the Chili-*S. rugosoannulata* and Rice-*S. rugosoannulata* rotations significantly improved the soil moisture before and after cultivating *S. rugosoannulata* and it is the highest in group Rice-*S. rugosoannulata* (*p* < 0.05). However, during the cultivating of *S. rugosoannulata*, there was no significant difference in soil moisture among the three groups (Fig. 2E) (*p* > 0.05).

### The soil rhizosphere microbial community diversity in different croplands

**Figure 3:**
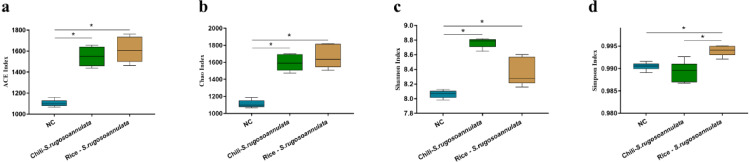
*α*-diversity of soil rhizosphere microorganisms after cultivating *S. rugosoannulata* in different croplands. (A) ACE index; (B) Chao index; (C) Shannon index; (D) Simpson index of soil rhizosphere microorganisms after cultivating *S. rugosoannulata*.

After cultivating *S. rugosoannulata*, Chili-*S. rugosoannulata*, and Rice-*S. rugosoannulata*, two soil rhizosphere microorganisms ACE index, Chao index, Shannon index, and Simpson index were significantly higher than the NC group (*p* < 0.05) (Figs. 3A–3D).

### The structure of soil rhizosphere microbial community in different croplands

**Figure 4:**
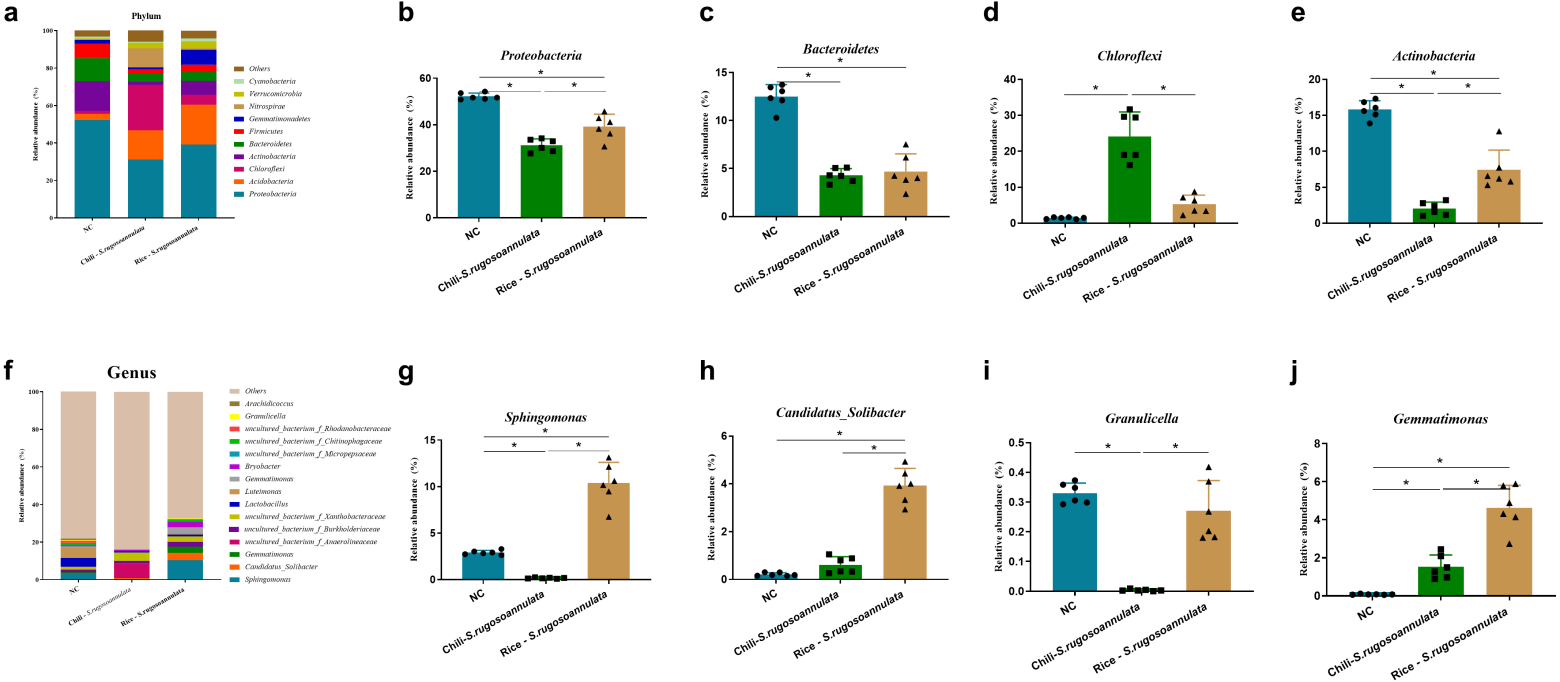
Rhizosphere bacterial community structure at phylum level after cultivating *S. rugosoannulata* in different croplands. (A) Relative abundance of rhizosphere microbiota at the phylum level after cultivating *S. rugosoannulata*; (B) percentage of *Proteobacteria* in each sample from the three groups after cultivating *S. rugosoannulata*; (C) percentage of *Bacteroidetes* in each sample from the three groups after cultivating *S. rugosoannulata*; (D) percentage of *Chloroflexi* in each sample from the three groups after cultivating *S. rugosoannulata*; (E) percentage of *Actinobacteria* in each sample from the three groups after cultivating *S. rugosoannulata*; (F) Relative abundance of rhizosphere microbiota at the genus level after cultivating *S. rugosoannulata*; (G) percentage of *Sphingomonas* in each sample from the three groups after cultivating *S. rugosoannulata*; (H) percentage of *Candidatus_Solibacter* in each sample from the three groups after cultivating *S. rugosoannulata*; (I) percentage of *Granulicella* in each sample from the three groups after cultivating *S. rugosoannulata*; (J) percentage of *Gemmatimonas* each sample from the three groups after cultivating *S. rugosoannulata*. Data are mean ± SD (*n* = 6) and analyzed by one-way ANOVA. *p < 0.05.

At the phylum level, after cultivating *S. rugosoannulata*, *Proteobacteria*, *Acidobacteria*, *Actinobacteria*, and *Chloroflexi* were the main microorganisms in the three groups, accounting for more than 70% of the total abundance (Fig. 4A). Compared with the NC group, the abundance of *Proteobacteria*, *Bacteroidetes*, and *Actinobacteriain* in the Chili-*S. rugosoannulata* group after cultivating *S. rugosoannulata* significantly decreased, while the abundance of *Chloroflexi* flora increased significantly (*p* < 0.05). Compared with the NC group, the abundance of *Bacteroidetes* and *Actinobacteriain* in the Rice-*S. rugosoannulata* group after cultivating *S. rugosoannulata* significantly decreased, while the abundance of *Chloroflexi* increased significantly (*p* < 0.05) (Figs. 4B–4E).

At the genus level, the bacterial community composition in the rhizosphere after cultivating *S. rugosoannulata* is shown in Fig. 4F. After cultivating *S. rugosoannulata*, compared with the NC group, the abundance of *Sphingomonas and Burkholderia-Caballeronia-Paraburkholderia* and *Granulicella* in the Chili-*S. rugosoannulata* group was significantly reduced, while the abundance of *Candidatus Solibacter* was significantly increased (*p* < 0.05). Rice-*S. rugosoannulata* group significantly increased the abundance of *Sphingomonas, C. Solibacter* and *Gemmatimonas* (*p* < 0.05) (Figs. 4G–4J).

### The structure of soil rhizosphere fungal community in different croplands

**Figure 5:**
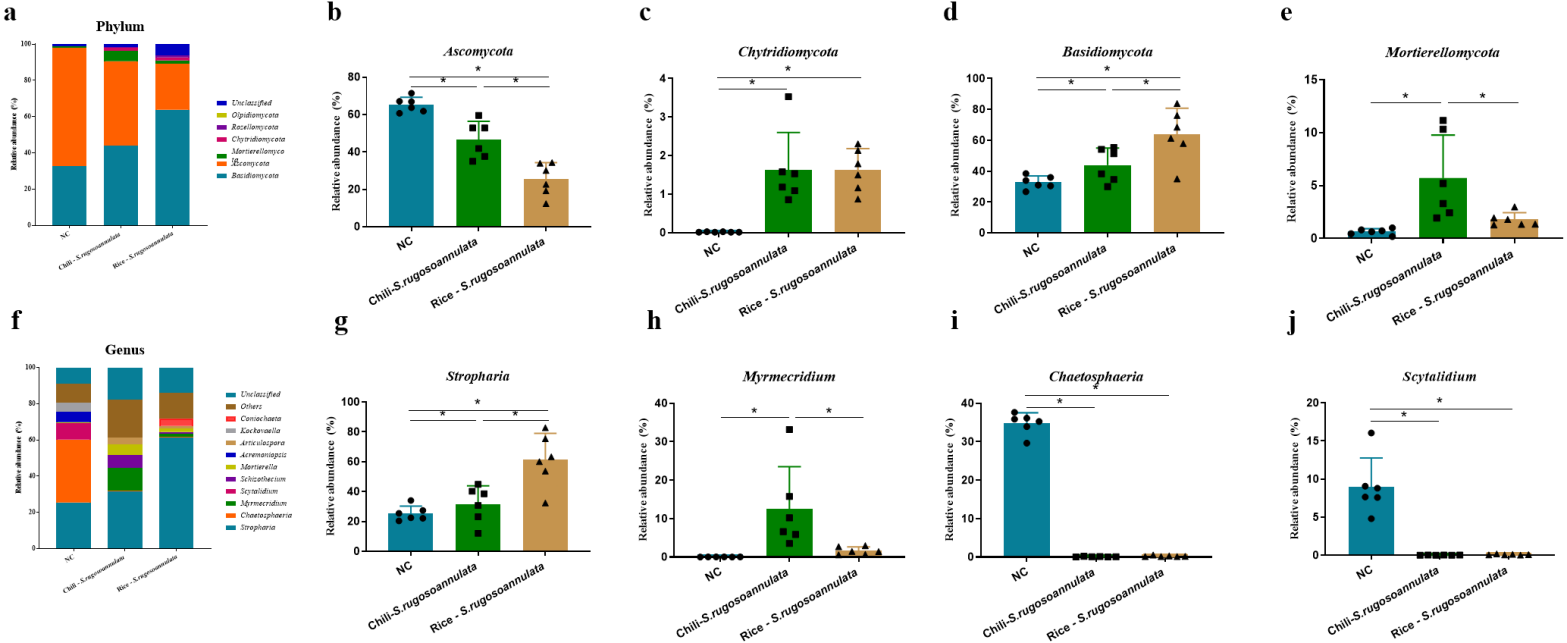
Rhizosphere fungal community structure at phylum level after cultivating *S. rugosoannulata* in different croplands. (A) Relative abundance of rhizosphere fungal at the genus level after cultivating *S. rugosoannulata*; (B) percentage of *Ascomycota* in each sample from the three groups after cultivating *S. rugosoannulata*; (C) percentage of *Chytridiomycota* in each sample from the three groups after cultivating *S. rugosoannulata*; (D) percentage of *Basidiomycota* in each sample from the three groups after cultivating *S. rugosoannulata*; (E) percentage of *Mortierellomycota* each sample from the three groups after cultivating *S. rugosoannulata*; (F) Relative abundance of rhizosphere fungal at the genus level after cultivating *S. rugosoannulata*; (G) percentage of *Stropharia* in each sample from the three groups after cultivating *S. rugosoannulata*; (H) percentage of *Myrmecridium* in each sample from the three groups after cultivating *S. rugosoannulata*; (I) percentage of *Chaetosphaeria* in each sample from the three groups after cultivating *S. rugosoannulata*; (J) percentage of *Scytalidium* each sample from the three groups after cultivating *S. rugosoannulata*. Data are mean ± SD (*n* = 6) and analyzed by one-way ANOVA. *p < 0.05.

At the phylum level, after cultivating *S. rugosoannulata*, *Basidiomycota*, *Ascomycota*, and *Mortierellomycota* were the main fungi in the three groups, accounting for more than 90% of the total abundance (Fig. 5A). Compared with the NC group, the abundance of *Ascomycota* in the Chili-*S. rugosoannulata* group after cultivating *S. rugosoannulata* significantly decreased, while the abundance of *Basidiomycota*, and *Mortierellomycota* increased significantly (*p* < 0.05). Compared with the NC group, the abundance of *Chytridiomycota* in the Chili-*S. rugosoannulata* group after cultivating *S. rugosoannulata* significantly decreased (*p* < 0.05). Compared with the NC group, the abundance of *Ascomycota* in the Rice-*S. rugosoannulata* group after cultivating *S. rugosoannulata* significantly decreased, while the abundance of *Chytridiomycota and Basidiomycota* increased significantly (*p* < 0.05) (Figs. 5B–5E).

At the genus level, the fungal community composition in the rhizosphere after cultivating *S. rugosoannulata* is shown in Fig. 5F. After cultivating *S. rugosoannulata*, compared with the NC group, the abundance of *Stropharia and Myrmecridium* in the Chili-*S. rugosoannulata* group was significantly increased, while the abundance of *Chaetosphaeria* and *Scytalidium* was significantly reduced (*p* < 0.05). Rice-*S. rugosoannulata* group significantly increased the abundance of *Stropharia,* but significantly decreased the abundance of *Chaetosphaeria* and *Scytalidium* (*p* < 0.05) (Figs. 5G–5J).

### Function prediction of bacteria and fungi produced in different croplands

**Figure 6:**
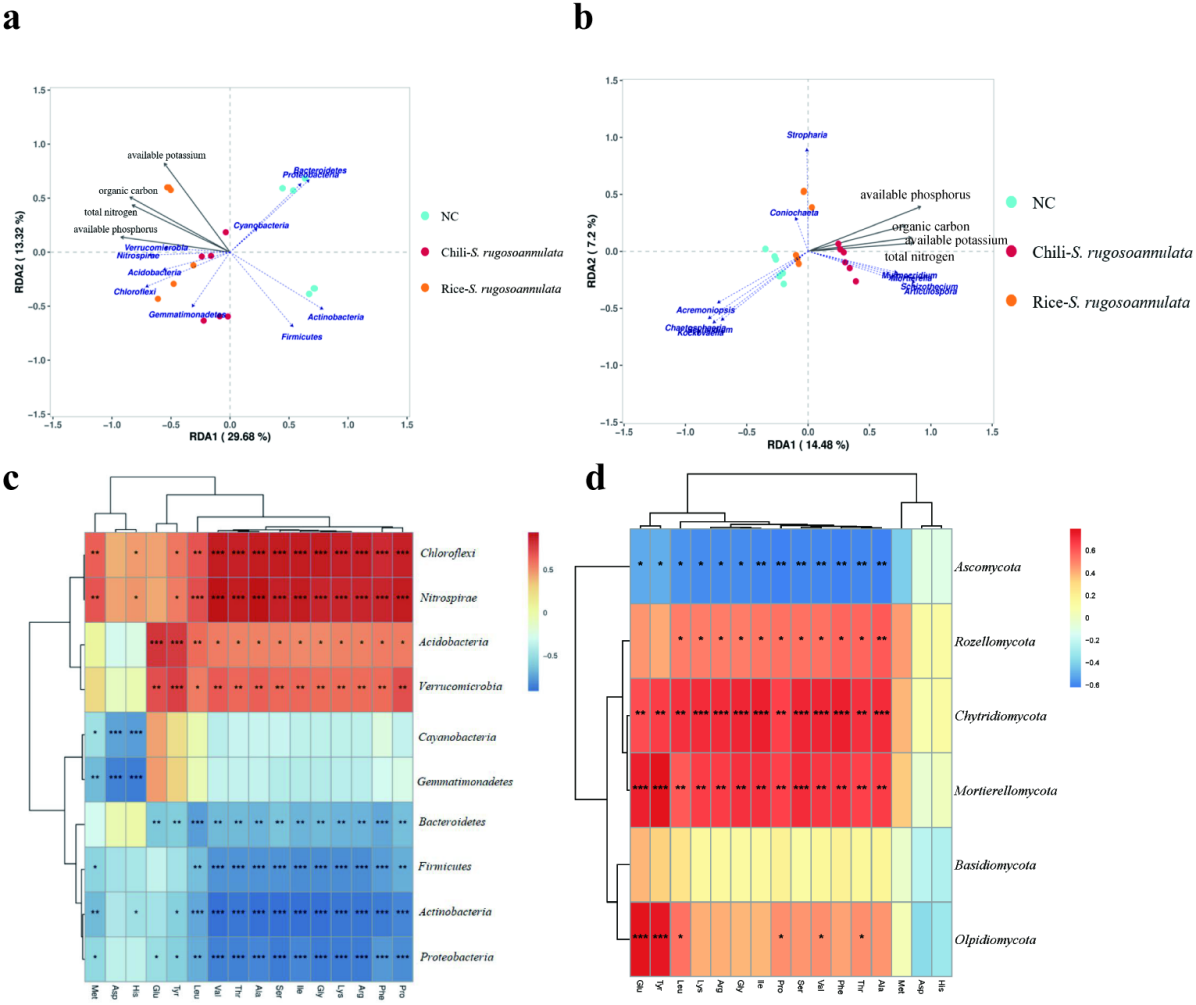
RDA analysis and correlation analysis of bacteria and fungi at the phylum level. (A) RDA analysis between soil rhizosphere bacteria and environmental factors. (B) RDA analysis between soil rhizosphere fungi and environmental factors. (C) Correlation analysis of soil rhizosphere bacteria at phylum level and *S. rugosoannulata* amino acid content *S. rugosoannulata.* (da) Correlation analysis of soil rhizosphere fungi at phylum level and *S. rugosoannulata* amino acid content *S. rugosoannulata*.

We found that soil organic carbon and available potassium had the greatest impact on the entire rotation process of cultivating *S. rugosoannulata* (Fig. 6A). We conducted a Pearson correlation analysis on the soil rhizosphere microorganisms at the phylum level and the amino acid content in *S. rugosoannulata*. We found that *Chloroflexi*, *Nitrospirae*, *Acidobacteria* and *Verrucomicrobia* are positively correlated with most amino acid content, while Bacteroidetes, Firmicutes, Actinobacteria and Proteobacteria are negatively correlated with most amino acid content (Fig. 6C). We used soil properties as environmental factors, and performed RDA analysis with soil rhizosphere fungi, and found that available phosphorus and available potassium had the greatest impact on the entire rotation process of cultivating *S. rugosoannulata* (Fig. 6B). We conducted a Pearson correlation analysis on the soil rhizosphere fungi at the phylum level and the amino acid content in *S. rugosoannulata*. We found that *Rozellomycota*, Chytridiomycota and *Mortierellomycota* are positively correlated with most amino acid content, while *Ascomycota* are negatively correlated with most amino acid content (Fig. 6D).

**Figure 7:**
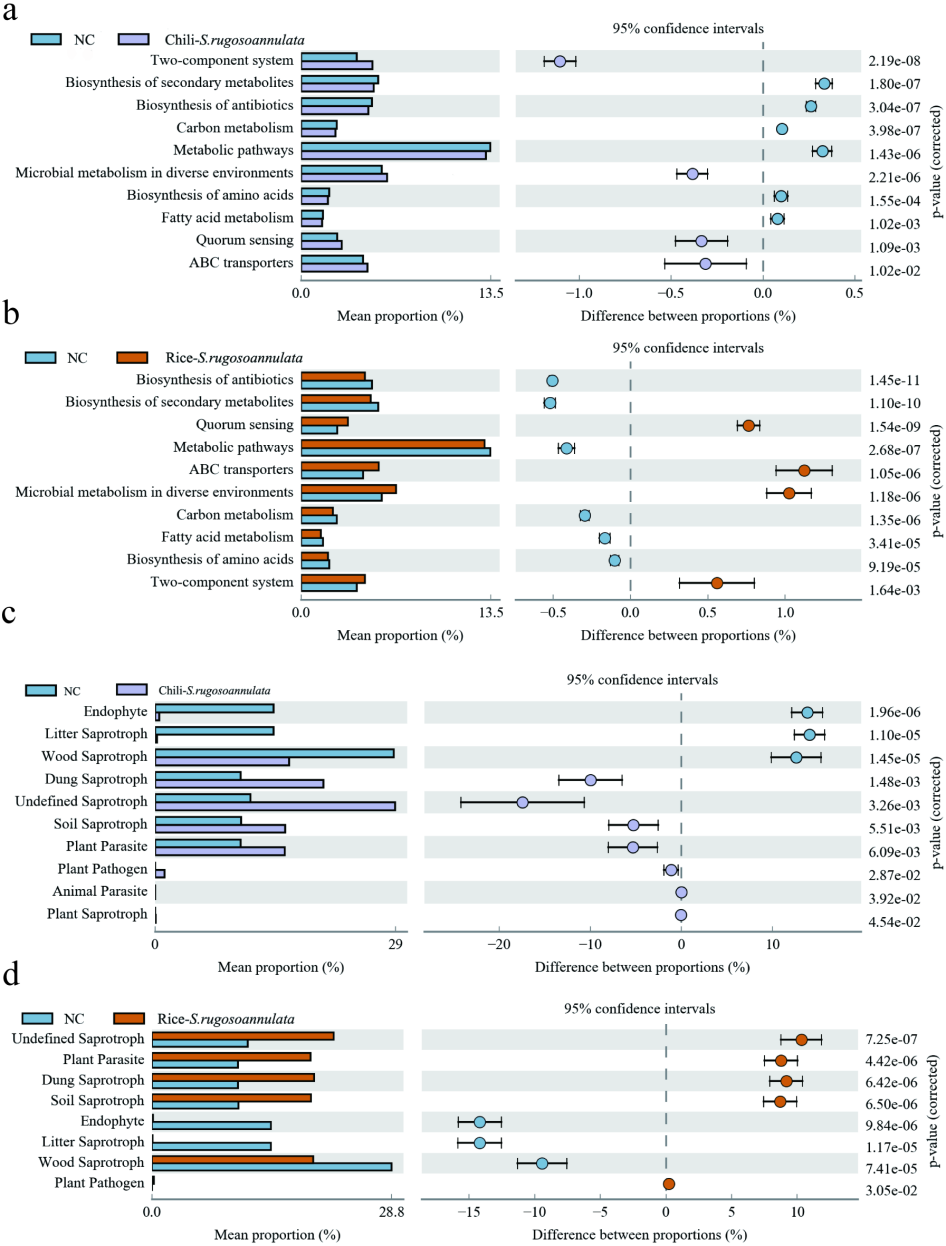
Prediction of microbial Tax4Fun functional genes in different croplands. (A) Differential bacterial functional genes between NC group and Chili-*S. rugosoannulata* group. (B) Differential bacterial functional genes between NC group and Rice-*S. rugosoannulata* group. (C) Differential fungi functional genes between NC group and Chili-*S. rugosoannulata* group. (D) Differential fungi functional genes between NC group and Rice-*S. rugosoannulata* group.

Finally, we found that compared with the NC group, the microbes in the Chili-*S. rugosoannulata* group (Fig. 7A) and the Rice-*S. rugosoannulata* group (Fig. 7B) were two-component system, Microbial metabolism in diverse environments, Quorum Sensing and ABC transporters related functional genes are significantly enriched. We conducted a predictive analysis of the FUNGuild between different groups and found that compared with the NC group, the fungi in the Chili-*S. rugosoannulata* group (Fig. 7C) and the Rice-*S. rugosoannulata* group (Fig. 7D) were Plant Parasite, Dung Saprotroph and Soil Saprotroph related functional genes are significantly enriched.

## Discussion

This experiment explored the effect of idle cropland of two crops, chili and rice, on the cultivation of *S. rugosoannulata*. The results showed that this cultivation method significantly increased the yield and amino acid content of *S. rugosoannulata* and significantly changed the soil microbial community structure.Generally speaking, soil properties have a great influence on mushroom cultivation (Liu et al., 2021a). Our experiments have proved that the two crop rotation ways have significantly increased the total nitrogen content of the soil and promoted the growth of *S. rugosoannulata*. From the results of our experiments, it was found that total nitrogen in the soil before cultivation of *S. rugosoannulata* was significantly elevated after the harvest of both crops compared to the open field. Therefore, we think that the increase of total nitrogen in soil may be a critical factor in promoting the growth of *S. rugosoannulata*, which was also verified in the experiments of Esmaeilzadeh-Salestani et al. (2022), nitrogen in the soil is taken up and transformed by ammonium transporters (AMTs) in plants and ultimately promote plant growth. This is consistent with prior research findings. Due to the rise in microbial variety in rice-planted fields, the ability of microbial nitrogen fixation has improved, resulting in an increase in the soil’s nitrogen content (Chakraborty & Islam, 2018). Also shown the same ability to manage soil microbes was pepper as a crop rotation. After planting pepper, the relative number of bacteria and fungi in the soil increased dramatically, resulting in an increase in the soil’s nitrogen concentration (Chen et al., 2021). Nitrogen in the soil can be taken and transformed by plant roots into amino acids, structural proteins, and genetic material, according to studies (Kerru et al., 2020; Bloom et al., 2012). This may explain why the amino acid content of *S. rugosoannulata* in our experiment increased dramatically under two rotation patterns. We also noticed that in addition to total nitrogen, the phosphorus content in soil was also significantly increased due to the two rotation modes. The rice ecosystem had a huge impact on the composition of soil microbial community structure, and the change of microbial community also changed the phosphorus cycle in soil (Xu et al., 2019). Increasing the soil’s phosphorus concentration can considerably boost spore production and the colonization rate of arbuscular mycorrhizal fungi (Lin et al., 2020). In our experimental results, both phosphorus levels were significantly higher in the soils of agricultural fields planted with chili and rice. It is due to this mechanism that a high nitrogen content in the soil can promote the growth of *S. rugosoannulata* and improve their quality and yield. Other than that, this may be due to the fact that different farming patterns regulates the structure of soil microbial community and promotes the conversion of phosphorus to available phosphorus. We discovered a significant increase in soil nutrient content in paddy fields, which may be a result of significant changes in microbial communities within the paddy fields’ anaerobic environment  (Breidenbach et al., 2016). In addition, the genome sequencing of *S. rugosoannulata* revealed that it possesses powerful bioremediation and lignin degradation capabilities, so the secondary metabolites that can be produced can significantly improve soil nutrient levels (Yang et al., 2022). It has been demonstrated that the used mushroom stock remaining after the fruiting bodies have been harvested adds many nutrients to the soil  (Gong et al., 2018).

There are many ways in which soil rhizosphere microorganisms play a role in promoting crop growth (Maddalwar et al., 2021), for example, fix nutrients in the soil, improve crop utilization, metabolize plant auxins and avoid pathogen infection (Vejan et al., 2016). According to our and previous research, the composition and structure of soil microorganisms are associated with soil nutrient levels (Mishra, Singh & Singh, 2022). Esmaeilzadeh-Salestani et al. (2022) experiment also proved our conjecture. Their research revealed that increasing soil organic carbon content was advantageous for increasing microbial diversity, whereas rotation was more favorable to preserving soil organic matter and maintaining microbial activity (Esmaeilzadeh-Salestani et al., 2021). Therefore, we investigated the effects of *S. rugosoannulata* on soil microbial structure under two rotation modes. Studies have found that *Chloroflexi* has the genomic potential of using formate, which can rapidly carry out the carbon cycle and improve soil organic matter and nutrients (McGonigle, Lang & Brazelton, 2020). Therefore, we speculate that in our experiment, it may be due to the increase in Chloroflexi abundance, thereby improving soil fertility and ultimately promoting the growth of *S. rugosoannulata*. *Sphingomonas* has outstanding performance in remediate soil pollution and promoting plant growth. Recent studies have found that *Sphingomonas* can produce plant growth hormones such as gibberellin and indole acetic acid, which can promote plant growth (Asaf et al., 2020). Our experiment also found that the abundance of *Sphingomonas* in agricultural soils after planting chili and rice significantly increased, which also verified that *Sphingomonas* can promote crop growth. *Gemmatimonas* is a phototrophic bacteria based on chlorophyll (Zeng et al., 2020). Light can provide more energy for its growth and metabolism, thereby increasing the content of soil organic matter (Koblížek et al., 2020). Similarly, *Gemmatimonas* was significantly increased in the Rice-*S. rugosoannulata* group, which promoted the growth of *S. rugosoannulata.* In our experiment, *Acidobacteria* increased significantly in the Rice-*S. rugosoannulat* group. Genomic studies have shown that *Acidobacteria* has the ability to convert inorganic nitrogen into organic compounds. In addition, *Acidobacteria* can secrete extracellular polysaccharides, which can change the properties of rhizosphere soil and promote crop nutrient absorption. It may be that Acidobacteria regulates soil properties and ultimately promotes the growth of *S. rugosoannulat.* By fungal sequencing analysis we found that the expression abundance of *Mortierella* in Chili-*S. rugosoannulata* and Rice-*S. rugosoannulataI* group significantly increase. Studies have shown that *Mortierella* can be used as a biological control agent to reduce the occurrence of root rot (Pimentel et al., 2020). In addition, *Cladosporium*, which is significantly increased in Chili-*S. rugosoannulata* and Rice-*S. rugosoannulataI* group, can metabolize and release two volatile organic compounds (2-methyl-butyraldehyde and 3-methyl-butyraldehyde), which can significantly enhance crop growth and roots development (Liu et al., 2021b). In our experiment, it also promoted the growth of *S. rugosoannulata*. After *S. rugosoannulata* was cultivated, the significant increase in *Stropharia* abundance in Chili-*S. rugosoannulata* and Rice-*S. rugosoannulataI* group should be due to the fact that the yield of *S. rugosoannulata* in the soil was significantly higher than that of the NC group. *Mycotoxins* is a fungus that can produce toxins (Rajachan et al., 2017).

Through gene function analysis of bacteria, we found that the two-component system, Microbial metabolism in diverse environments, was significantly enriched in Chili-*S. rugosoannulata* and Rice-*S. rugosoannulata* groups. Bahn et al. (2006) find the two-component system can cascades control fundamental cellular functions related to fungal growth. Most two-component system-related signaling proteins are essential for fungal growth (Virginia et al., 2000). Microbial metabolism-related genes regulate bacterial metabolism and promote bacterial production of growth-promoting factors to regulate crop growth (Larsbrink et al., 2017). This also indicates that the soil microbial community structure was greatly regulated after cultivation of *S. rugosoannulata*, and that the microbial growth-promoting function was improved. Thus, we believe that mushroom cultivation in winter on vacant cropland can not only improve its quality and yield, but also increase soil microbial diversity, improve soil fertility, and lay a good foundation for a new round of crops in the coming year.

## Conclusions

In this study, we systematically investigated changes in soil microbial communities in three cropping patterns: blank cropland, Chili-*S. rugosoannulata* and Rice-*S. rugosoannulata*. The results showed that the two crop rotation modes, Chili-*S. rugosoannulata* and Rice-*S. rugosoannulata*, significantly increased the yield, quality, and amino acid content of *S. rugosoannulata*, improved soil quality. Notably, S. rugosoannulata cultivation significantly increased the abundance of beneficial microorganisms such as *Chloroflexi*, *Cladosporium* and *Mortierella* and reduce the abundance of *Botryotrichumin* and *Archaeorhizomyces*. This leads us to believe that *S. rugosoannulata* cultivation in cropland can improve soil properties, regulate the community structure of soil microorganisms, increase the expression abundance of beneficial organisms and ultimately improve the *S. rugosoannulata* yield and lay a good foundation for a new round of crops after this edible mushroom cultivation.

##  Supplemental Information

10.7717/peerj.14130/supp-1 Data S1 Raw data on yield, soil properties and amino acid content (Figures 1, 2 and Table 1)

## Additional Information and Declarations

### Data Availability

The following information was supplied regarding data availability: The raw sequences are available at NCBI Sequence Read Archive: PRJNA787767.

## References

[ref-1] Asaf S, Numan M, Khan AL, Al-Harrasi A (2020). Sphingomonas: from diversity and genomics to functional role in environmental remediation and plant growth. Critical Reviews in Biotechnology.

[ref-2] Bafokuzara ND (1983). Influence of six vegetable cultivars on reproduction of Meloidogyne javanica. Journal of Nematology.

[ref-3] Bahn Y-S, Kojima K, Cox GM, Heitman J (2006). A unique fungal two-component system regulates stress responses, drug sensitivity, sexual development, and virulence of Cryptococcus neoformans. Molecular Biology of the Cell.

[ref-4] Bloom AJ, Randall L, Taylor AR, Silk WK (2012). Deposition of ammonium and nitrate in the roots of maize seedlings supplied with different nitrogen salts. Journal of Experimental Botany.

[ref-5] Bolger AM, Lohse M, Usadel B (2014). Trimmomatic: a flexible trimmer for Illumina sequence data. Bioinformatics.

[ref-6] Bray RH, Kurtz LT (1945). Determination of total, organic, and available forms of phosphorus in soils. Soil Science.

[ref-7] Breidenbach B, Blaser MB, Klose M, Conrad R (2016). Crop rotation of flooded rice with upland maize impacts the resident and active methanogenic microbial community. Environmental Microbiology.

[ref-8] Chakraborty A, Islam E (2018). Temporal dynamics of total and free-living nitrogen-fixing bacterial community abundance and structure in soil with and without history of arsenic contamination during a rice growing season. Environmental Science and Pollution Research.

[ref-9] Chen W, Guo X, Guo Q, Tan X, Wang Z (2021). Long-term chili monoculture alters environmental variables affecting the dominant microbial community in rhizosphere soil. Frontiers in Microbiology.

[ref-10] Ciavatta C, Govi M, Antisari LV, Sequi P (1991). Determination of organic carbon in aqueous extracts of soils and fertilizers. Communications in Soil Science and Plant Analysis.

[ref-11] Dane JH, Topp GC, Campbell GS (2002). Methods of soil analysis.

[ref-12] De la Fuente ME, Beyer DM, Rinker DL (1998). First Report of Trichoderma harzianum Biotype Th4, on Commercial Button Mushrooms in California. Plant Disease.

[ref-13] Edgar RC (2013). UPARSE: highly accurate OTU sequences from microbial amplicon reads. Nature Methods.

[ref-14] Edgar RC, Haas BJ, Clemente JC, Quince C, Knight R (2011). UCHIME improves sensitivity and speed of chimera detection. Bioinformatics.

[ref-15] Eremeev V, Talgre L, Kuht J, Maeorg E, Esmaeilzadeh-Salestani K, Alaru M, Loit E, Runno-Paurson E, Luik A (2020). The soil microbial hydrolytic activity, content of nitrogen and organic carbon were enhanced by organic farming management using cover crops and composts in potato cultivation. Acta Agriculturae Scandinavica Section B.

[ref-16] Esmaeilzadeh-Salestani K, Bahram M, Seraj RGM, Gohar D, Tohidfar M, Eremeev V, Talgre L, Khaleghdoust B, Mirmajlessi S, Luik A, Loit E (2021). Cropping systems with higher organic carbon promote soil microbial diversity. Agriculture Ecosystems & Environment.

[ref-17] Esmaeilzadeh-Salestani K, Bahraseman MRS, Tohidfar M, Khaleghdoust B, Keres I, Mottus A, Loit E (2022). Expression of AMT1;1 and AMT2;1 is stimulated by mineral nitrogen and reproductive growth stage in barley under field conditions. Journal of Plant Nutrition.

[ref-18] Gong S, Chen C, Zhu J, Qi G, Jiang S (2018). Effects of wine-cap *Stropharia* cultivation on soil nutrients and bacterial communities in forestlands of northern China. PeerJ.

[ref-19] Grimm D, Wösten HAB (2018). Mushroom cultivation in the circular economy. Applied Microbiology and Biotechnology.

[ref-20] Hahm MS, Son J-S, Kim B-S, Ghim S-Y (2017). Comparative study of rhizobacterial communities in pepper greenhouses and examination of the effects of salt accumulation under different cropping systems. Archives of Microbiology.

[ref-21] Hamza MA, Anderson W (2005). Soil compaction in cropping systems: a review of the nature, causes and possible solutions. Soil and Tillage Research.

[ref-22] Hao WL, Liang Y-L, Wu X, Lin X-J, Zhu Y-L, Luo A-R (2011). Soil respiration variations in winter wheat field in different previous crops and its influencing factors. Huan Jing Ke Xue.

[ref-23] Hou PF, Chien C-H, Chiang-Hsieh Y-F, Tseng K-C, Chow C-N, Huang H-J, Chang W-C (2018). Paddy-upland rotation for sustainable agriculture with regards to diverse soil microbial community. Scientific Reports.

[ref-24] Jackson ML (2005). Soil chemical analysis: advanced course.

[ref-25] Kerru N, Gummidi L, Maddila S, Gangu KK, Jonnalagadda SB (2020). A review on recent advances in nitrogen-containing molecules and their biological applications. Molecules.

[ref-26] Koblížek M, Dachev M, Bina D, Nupur, Piwosz K, Kaftan D (2020). Utilization of light energy in phototrophic Gemmatimonadetes. Journal of Photochemistry and Photobiology B.

[ref-27] Koutrotsios G, Danezis G, Georgiou C, Zervakis GI (2020). Elemental content in pleurotus ostreatus and cyclocybe cylindracea mushrooms: correlations with concentrations in cultivation substrates and effects on the production process. Molecules.

[ref-28] Kuht J, Eremeev V, Talgre L, Alaru M, Loit E, Maeorg E, Esmaeilzadeh-Salestani K, Luik A (2019). Changes in the soil microbial hydrolytic activity and the content of organic carbon and total nitrogen by growing spring barley undersown with red clover in different farming systems. Agriculture-Basel.

[ref-29] Kumar R, Mishra JS, Rao KK, Mondal S, Hazra KK, Choudhary JS, Hans H, Bhatt BP (2020). Crop rotation and tillage management options for sustainable intensification of rice-fallow agro-ecosystem in eastern India. Scientific Reports.

[ref-30] Langille MG, Zaneveld J, Caporaso JG, McDonald D, Knights D, Reyes JA, Clemente JC, Burkepile DE, Thurber RLV, Knight R, Beiko RG, Huttenhower C (2013). Predictive functional profiling of microbial communities using 16S rRNA marker gene sequences. Nature Biotechnology.

[ref-31] Larsbrink J, Tuveng TR, Pope PB, Bulone V, Eijsink VGH, Brumer H, McKee LS (2017). Proteomic insights into mannan degradation and protein secretion by the forest floor bacterium Chitinophaga pinensis. Journal of Proteomics.

[ref-32] Lin C, Wang Y, Liu M, Li Q, Xiao W, Song X (2020). Effects of nitrogen deposition and phosphorus addition on arbuscular mycorrhizal fungi of Chinese fir (Cunninghamia lanceolata). Scientific Reports.

[ref-33] Lipiec J, Usowicz B, Klopotek J, Turski M, Frac M (2021). Effects of application of recycled chicken manure and spent mushroom substrate on organic matter, acidity, and hydraulic properties of sandy soils. Materials.

[ref-34] Liu Y, Hu C-F, Feng X, Cheng L, Ibrahim SA, Wang C-T, Huang W (2020). Isolation, characterization and antioxidant of polysaccharides from *Stropharia rugosoannulata*. International Journal of Biological Macromolecules.

[ref-35] Liu D, Wang Y, Zhang P, Yu F, Perez-Moreno J (2021a). Macrofungi cultivation in shady forest areas significantly increases microbiome diversity, abundance and functional capacity in soil furrows. Journal of Fungi.

[ref-36] Liu N, Zhou L, Fang J, Jiang H, Liu G (2021b). Effects of IQW and IRW on inflammation and gut microbiota in etec-induced diarrhea. Mediators of Inflammation.

[ref-37] Maddalwar S, Nayaka KK, Kumar M, Singh L (2021). Plant microbial fuel cell: opportunities, challenges, and prospects. Bioresource Technology.

[ref-38] Magoč T, Salzberg SL (2011). FLASH: fast length adjustment of short reads to improve genome assemblies. Bioinformatics.

[ref-39] McGonigle JM, Lang SQ, Brazelton WJ (2020). Genomic evidence for formate metabolism by chloroflexi as the key to unlocking deep carbon in lost city microbial ecosystems. Applied and Environmental Microbiology.

[ref-40] Mishra A, Singh L, Singh D (2022). Unboxing the black box-one step forward to understand the soil microbiome: a systematic review. Microbial Ecology.

[ref-41] Pejin B, Tesanovic K, Jakovljevic D, Kaisarevic S, Sibul F, Raseta M, Karaman M (2019). The polysaccharide extracts from the fungi *Coprinus comatus* and *Coprinellus truncorum* do exhibit AChE inhibitory activity. Natural Product Research.

[ref-42] Peltonen-Sainio P, Jauhiainen L, Lehtonen H (2016). Land use, yield and quality changes of minor field crops: is there superseded potential to be reinvented in Northern Europe?. PLOS ONE.

[ref-43] Pimentel MF, Arnao E, Warner AJ, Subedi A, Rocha LF, Srour A, Bond JP, Fakhoury AM (2020). Trichoderma isolates inhibit fusarium virguliforme growth, reduce root rot, and induce defense-related genes on soybean seedlings. Plant Disease.

[ref-44] Rajachan OA, Kanokmedhakul K, Soytong K, Kanokmedhakul S (2017). Mycotoxins from the fungus botryotrichum piluliferum. Journal of Agricultural and Food Chemistry.

[ref-45] Tesanovic K, Pejin B, Sibul F, Matavulj M, Raseta M, Janjusevic L, Karaman M (2017). A comparative overview of antioxidative properties and phenolic profiles of different fungal origins: fruiting bodies and submerged cultures of *Coprinus comatus* and *Coprinellus truncorum*. Journal of Food Science and Technology-Mysore.

[ref-46] Vejan P, Abdullah R, Khadiran T, Ismail S, Boyce AN (2016). Role of plant growth promoting rhizobacteria in agricultural sustainability-a review. Molecules.

[ref-47] Virginia M, Appleyard CL, McPheat WL, Stark MJ (2000). A novel ‘two-component’ protein containing histidine kinase and response regulator domains required for sporulation in *Aspergillus nidulans*. Current Genetics.

[ref-48] Wu J, Fushimi K, Tokuyama S, Ohno M, Miwa T, Koyama T, Yazawa K, Nagai K, Matsumoto T, Hirai H, Kawagishi H (2011). Functional-food constituents in the fruiting bodies of *Stropharia rugosoannulata*. Bioscience, Biotechnology, and Biochemistry.

[ref-49] Wu J, Tokuyama S, Nagai K, Yasuda N, Noguchi K, Matsumoto T, Hirai H, Kawagishi H (2012). Strophasterols A to D with an unprecedented steroid skeleton: from the mushroom *Stropharia rugosoannulata*. Angewandte Chemie International Edition.

[ref-50] Xu M, Gao P, Yang Z, Su L, Wu J, Yang G, Zhang X, Ma J, Peng H, Xiao Y (2019). Biochar impacts on phosphorus cycling in rice ecosystem. Chemosphere.

[ref-51] Yan Q-X, Huang M-X, Sun P, Cheng S-X, Zhang Q, Dai H (2020). Steroids, fatty acids and ceramide from the mushroom *Stropharia rugosoannulata* Farlow apud Murrill. Biochemical Systematics and Ecology.

[ref-52] Yang Y, Meng G, Ni S, Zhang H, Dong C (2022). Genomic analysis of *Stropharia rugosoannulata* reveals its nutritional strategy and application potential in bioremediation. Journal of Fungi.

[ref-53] Yun Y, Lu Z, Yang J, Liang T, Xiao G, Qiao Y, Liu Y (2021). Electrochemical analysis of specific catalase activity during development of Aspergillus flavus and its correlation with aflatoxin B1 production. Food Chemistry.

[ref-54] Zeng Y, Nupur, Wu N, Madsen AM, Chen X, Gardiner AT, Koblizek M (2020). Gemmatimonas groenlandica sp. nov. Is an aerobic anoxygenic phototroph in the phylum gemmatimonadetes. Frontiers in Microbiology.

[ref-55] Zhou W, Lv T-F, Chen Y, Westby AP, Ren W-J (2014). Soil physicochemical and biological properties of paddy-upland rotation: a review. Scientific World Journal.

